# Uterine Artery Embolization as Nonsurgical Treatment of Uterine Myomas

**DOI:** 10.5402/2011/489281

**Published:** 2011-11-23

**Authors:** Strinic Tomislav, Maskovic Josip, Cambi Sapunar Liana, Vulic Marko, Jukic Marko, Radic Ante, Jelcic Dzenis, Grandic Leo, Stipic Ivica, Tandara Marijan, Kristina Situm

**Affiliations:** ^1^Department of Gynecology and Obstetrics, University Hospital, Spincica 1, 21000 Split, Croatia; ^2^School of Medicine, Split University, Soltanska 2, 2100 Split, Croatia; ^3^Radiology Institute, University Hospital, Spincica 1, 2100 Split, Croatia

## Abstract

The purpose of this study was to evaluate safety, efficacy or complications of uterine artery embolization (UAE). Patients with symptomatic uterine fibroids (*n* = 157) were treated by selective bilateral UAE using 350–500 **μ**m sized polyvinyl alcohol particles. Bilateral UAE was successful in 152 (96.8%) cases. Baseline measures of clinical symptoms and MRI taken before the procedure were compared to those taken 3, 6, and 12 months after embolotherapy. Also, complications and outcomes were analyzed after procedure. All patients had an uneventful recovery and were able to return to normal activity within two weeks of embolization. After the procedure, most patients experienced crampy pelvic pain, of variable intensity, which was well managed with the standard analgesia protocol. Five (3%) of participants had persisting amenorrhea after procedure. None reported any new gynecologic or medical problem during the follow-up period. There were no deaths and no major permanent injuries. Reductions in mean uterine volume were 61% (*P* < 0.01) and in dominant fibroid volume 66% (*P*≤0.01). The follow-up showed significant improvement of bleeding. In conclusion, uterine artery embolization is a successful, minimal invasive treatment of uterine fibroids that preserves the uterus, had minimal complications, and requires short hospitalization and recovery.

## 1. Introduction

Uterine fibroids (leiomyomata or myomas) are benign tumours of smooth muscle cells and fibrous connective tissue that develop within the walls of the uterus. They are the most common gynaecological problem experienced by women, being of clinical significance in 20–40% of women of childbearing age. The true prevalence of uterine fibroids is unknown because the majority of these tumours are asymptomatic. Traditionally, symptomatic fibroids have been treated with myomectomy or hysterectomy performed by laparotomy [1, 2].

 Over the last few years, a variety of new treatment approaches have become available to women with symptomatic fibroids. Undoubtedly the most significant therapeutic innovation has been the advent of uterine artery embolization (UAE) as a form of nonsurgical management. Embolization is also an excellent option for patients who will not accept blood transfusions and for those who are severely anaemic and require immediate intervention [3, 4]. Careful preprocedure evaluation is essential to exclude pregnancy and genital tract malignancy. Other absolute contraindications include comorbidities that may increase the risk for infectious complications (e.g., pelvic inflammatory disease or active genitourinary infection), the presence of an adnexal mass, and conditions that contraindicate any endovascular procedure (e.g., reduced immune status, severe coagulopathy, severe contrast medium allergy, or impaired renal function). The desire to avoid a hysterectomy under any circumstances is also an absolute contraindication to UAE. There are no restrictions to the size and number of fibroids that can be treated with UAE. Anatomic exclusion criteria only include submucosal fibroids that may be effectively treated with hysteroscopic resection and pedunculated subserosal fibroids with a narrow stalk because of the potential risk and complications from infarction of the stalk and subsequent fibroid detachment from the uterus [7]. The aim of our study was to evaluate the safety and efficacy of uterine fibroid embolization in patients with symptomatic uterine fibroids.

## 2. Materials and Methods

The study cohort consisted of 157 premenopausal women with ultrasound documented symptomatic fibroids. All were consecutively selected from women presenting for evaluation for uterine artery embolisation at the Department of Obstetrics and Gynecology of the University Hospital Split, Croatia, between May 1999 and November 2006. According to our existing protocol, patients were considered suitable for UAE if they had single or multiple myomas causing symptoms (namely, heavy menstrual bleeding and bulk related symptoms, which included pelvic pain and pressure effects) sufficiently severe to warrant hysterectomy or myomectomy and wished to avoid surgery. Eligibility was not restricted by age, fibroid size, location, or previous surgery. Although women desiring children were not excluded from the study, they were further informed of the uncertain effects of UAE on conception or carrying to full term. Exclusion criteria also included patients with pregnancy, active pelvic inflammatory disease, renal insufficiency, undiagnosed pelvic mass, or urogenital infection. A detailed gynecologic history was obtained from each patient, followed by a detailed description of the procedure including a discussion of its potential risks. This study was approved by the Hospital Ethic Committee, with written informed consent obtained from each participant at the time of enrollment.

During preprocedural testing, each patient underwent venous blood sampling (complete blood count, blood urea nitrogen, creatinine, prothrombin time) and magnetic resonance imaging (MRI) of the uterus. Measurements of the uterus and volume of the dominant fibroid were calculated. All the patients were admitted to the department of gynecology the day before the procedure. They completed the questionnaire including information on demographics and medical and gynecologic history. All procedures were performed by the same interventional radiologist according to the same procedure protocol. Under local anesthesia, vascular access was obtained with 5F catheter via the right femoral artery and aortic bifurcation to the contralateral internal iliac artery. Digital angiography was performed to identify the origin of the uterine artery, and thereafter, the left uterine artery was catheterized with coaxial 3F microcatheter. The tip of the microcatheter was placed in the distal third of the left uterine artery, and 350–500 *μ*m sized polyvinyl alcohol particles (Ivalon, Nycomed, Paris) were injected until there was complete stasis of flow. After confirming the presence of a stagnant column of contrast in the left uterine artery, the right uterine artery was catheterized in similar fashion and embolized. The procedure is completed when there is no flow in either uterine artery. All catheters were removed and groin pressure was applied for 10 to 15 minutes, thus completing the procedure. The goal of the therapy was to occlude the uterine artery branches that supply only the fibroid tumors and spare normal myometrial vessels.The arteriograms obtained after embolization revealed complete occlusion of the branches supplying the fibroids. After the procedure, patients were kept in hospital for 24–48 hours for further observation hematoma formation at the arterial puncture site and pain control. The patients had received intravenous medications for nausea, vomiting or pain control. The majority of the patients left the hospital next day after the procedure. They completed outcome questionnaires following their treatment. All patients with successful procedures were evaluated at 3, 6, and 12 months after embolization with gynecologic examination, magnetic resonance imaging, and questionnaire. They were asked whether their symptoms resolved completely, improved, remained unchanged, or deteriorated. Furthermore, they were asked about their satisfaction with the procedure. Measurements of the uterus and volume of the dominant fibroid were calculated. The percent volume reduction was calculated for each patient. Symptom change and patient satisfaction were classified as markedly improved, moderately improved, slightly improved, unchanged, and worse. 

 Descriptive statistics, including means and ranges, were calculated for dominant fibroid and uterine volumes, demographic and clinical characteristics. Differences in dominant fibroid and uterine volumes before and after UAE were analyzed with Student's paired *t*-test. Statistical significance was set at a *P*  value < 0.05.

## 3. Results and Discussion

Baseline patient characteristics are summarized in Table 1. There were 157 patients included in the study, but bilateral UAE was successful in 152 (96.8%) cases. Five (3.2%) procedures were technically unsuccessful, four because of malformed vessels and one of them had allergic reaction to contrast medium. We excluded from statistical data processing unsuccessful procedures. 

The mean procedure time was 37 minutes, range 25–81 minutes. All of the patients went home within the first week after the procedure (range 1–6 days). The majority of women went home the day after UAE (mean hospitalization was 1.4 days).

Table 2 summarizes complications of the embolization. After the procedure, most patients experienced crampy pelvic pain, of variable intensity, which was well managed with the standard analgesia protocol (narcotics and nonsteroidal anti-inflammatory drugs). Some of the participants had nausea, and only few of them had vomiting. Both symptoms were successfully cured with antiemetics. All patients had an uneventful recovery and were able to return to normal activity within two weeks of embolization. Five (3%) of participants had persisting amenorrhea after procedure. All of them were older than 45 years. None reported any new gynecologic or medical problem during the follow-up period. There were no deaths and no major permanent injuries. No patients were lost to follow-up. 

Rate of uterine regression and dominant fibroid volume determined by magnetic resonance scanning 3, 6, and 12 months after procedure is shown in Table 3. Median uterine volume decreased by 38%, 57%, and 61% after 3, 6, and 12 months after embolotherapy, respectively. Comparison of the regression of preprocedural and final uterine volume revealed statistical significance (*P* < 0.01). Median dominant fibroid volume decreased by 46%, 61%, and 66% after 3, 6, and 12 months from preprocedure values, respectively. The quantum regression of pretreatment to final dominant fibroid volume also revealed statistical significance (*P* < 0.01).

Table 4 shows that patient satisfaction with UAE treatment was paralleled with symptomatic outcome. Moderate-to-great satisfaction was expressed by 92 percent of patients. All women reported resumption of regular menses except five (3%) with persisting amenorrhea. 

 Comparing UAE and surgery it was apparent that hysterectomy involves major surgery and has associated risks. Women stay in hospital for on average 6 days and then may take up to 3 months to recuperate. However, once the uterus is removed, there is no recurrence of fibroid-related symptoms. UAE is a minimally invasive technique with a short-term complication rate, and the great majority of women remain in hospital overnight to enable appropriate pain management [5, 6]. Crampy postembolization pain occurs frequently. Pain usually peaks on the first day following the procedure, but occasionally on the second day, and rarely on the third day postprocedure. Resolution of pain can be expected in 1 week. Pain syndromes lasting longer than 2 weeks are rare. The pain is probably due to the ischemia produced by the embolization procedure. Strong analgesics and, particularly, patient controlled analgesia (during hospitalization) are extremely helpful during this period [3–8]. We believe that an overnight admission is desirable, but others have advocated embolization on an outpatient basis [3–8]. Premature menopause has been documented in 1% to 2% of patients after UAE and is believed to result from nontarget embolization of the ovaries via the collateral bed between the ovarian and the uterine arteries. This is a risk that must be considered in a premenopausal woman who desires future fertility. All authors who have published a series of any size have reported essentially similar outcome statistics [9–13]. 

 Major complications consist of infection, pyometra, terrible pain, and death. Two deaths from uterine infection and overwhelming sepsis have also been reported after UAE. In addition to two deaths from septic shock [14, 15], other three deaths following UAE have thus far been reported, one from pulmonary embolism [16] and two from uncertain causes [13], in more than 100 000 procedures performed worldwide. A relatively common complication of UAE is vaginal expulsion of an infarcted fibroid, with a reported rate of up to 10% [9, 13, 17]. This complication is more frequently seen in patients with submucosal fibroids or intramural fibroids with a submucosal component. Expulsion most often occurs within 6 months after the procedure. In most cases, the infarcted fibroid is expelled spontaneously and no additional treatment is necessary [9, 13, 17]. 

Minor complications consist of groin hematoma, allergic reaction, and pain. Our inital experience parallels that of others [3–17]. Clinical response has been high. Eighty percent to 90% of patients embolized have reported significant improvements in menorrhagia, bulk-related symptoms, or both. In addition to the improvement in symptoms, patients have experienced significant volume reductions. Reduction in overall uterine volume averages 50% at 3 months after embolization and 67% at six months after embolization. Individual myomas showed average volume reductions at 60% to 65%, with a significant number of myomas becoming no longer visible at follow-up study [3–17]. Our study shows similar results. There have been no reports of reduced fertility after occlusion of the uterine vessels. At the same time there are numerous reports of successful pregnancies after UAE [12]. 

In conclusion, after tens of thousands of successfully performed UAE worldwide, it is proved that this method is an effective alternative to surgery. UAE is a successful, minimal invasive treatment of myomas that preserves the uterus and requires shorter hospitalization and recovery times than surgery. The complication rate is low, and the results are rapid and impressive. In the near future, embolization might replace conventional medical and surgical treatments of uterine fibroids. The results of this study indicate that this procedure might be recommended as a primary treatment for young patients with fibroids who wish to preserve, or enhance, their fertility.

## Figures and Tables

**Table 1:** Characteristics of patients with successful procedures (*N* = 152).

	Mean ± SD	Range
Age (years)	42.9 ± 4.1	36–51
Weight (kg)	67.7 ± 4.7	55–87
Height (cm)	168.5 ± 3.2	157–181
Parity (number)	2.2 ± 0.4	1–4
Procedure time (min.)	37.0 ± 4.3	25–81
Duration of hospitalization (days)	1.4 ± 0.5	1–6

**Table 2:** Complications after uterine artery embolization (*N* = 152).

	*N*	(%)
Moderate pain	21	(14)
Severe pain	7	(5)
Fever after procedure	14	(9)
Fibroid expulsion	2	(1)
Transient amenorrhea	7	(5)
Persisting amenorrhea	5	(3)

**Table 3:** Rate of regression of uterine and dominant fibroid volume determined by MRI (*N* = 152).

	Follow-up (months)				
	0	3	6	12	*P* level*

Uterine volume (cm^3^)	860	534 (−38%)	370 (−57%)	335 (−61%)	<0.01
Dominant fibroid volume (cm^3^)	385	214 (−46%)	155 (−61%)	134 (−66%)	<0.01

*t-test.

**Table 4:** Experience/satisfaction of the patients (*N* = 152).

	*N*	(%)
Markedly improved	108	(71)
Moderately improved	32	(21)
Slightly improved	9	(6)
Unchanged	3	(2)
Worse	0	
